# Reliability and validity of the Korean version of the MacNew heart disease Health-related Quality of Life questionnaire

**DOI:** 10.1186/s12955-021-01832-7

**Published:** 2021-08-14

**Authors:** Sun Hyoung Bae, Myeong-Ho Yoon, Jin-Hee Park

**Affiliations:** 1 College of Nursing·Research Institute of Nursing Science, Ajou University, 164, World cup-Ro, Yeongtong-Gu, Suwon, 16499 Korea; 2 Department of Cardiology, Ajou University Medical Center, Suwon, South Korea

**Keywords:** Reliability, Validity, Translation, Coronary artery disease, Health-related Quality of Life

## Abstract

**Background:**

Myocardial infarction and unstable angina are prevalent in Korea. The MacNew Heart Disease health-related quality of life questionnaire is a widely used patient-reported outcome measure for patients with heart disease in several countries. In this study, we tested the validity and reliability of the Korean version of the MacNew (K-MacNew).

**Methods:**

Participants were 200 patients who had experienced unstable angina or myocardial infarction, and were recruited from a tertiary hospital in Korea. The K-MacNew was developed using forward–backward translation techniques. Construct validity (including discriminative validity), concurrent validity, and internal consistency reliability of the K-MacNew were assessed. Discriminative validity was assessed by examining the between-group differences in the K-MacNew scores according to functional capacity, anxiety, and depression levels. Concurrent validity was examined by correlating the K-MacNew dimensions with the physical and mental health domains of the 36-item Short Form Health Survey Instrument (SF-36).

**Results:**

Factor analysis results of the K-MacNew demonstrated a three-factor structure (emotional, physical, and social) that explained 57.92% of the variance. Significant differences in the K-MacNew scores were observed according to patients’ functional capacity, anxiety, and depression levels. The SF-36 physical health domain score showed a moderate positive correlation with the physical dimension score of the K-MacNew (r = 0.517, *P* < 0.001), and the SF-36 mental health domain score showed a strong positive correlation with the emotional dimension of K-MacNew (r = 0.745, *P* < 0.001). The K-MacNew showed good internal consistency, with a Cronbach’s α of 0.947 for the global scale.

**Conclusion:**

The K-MacNew demonstrated good reliability and validity for use as a patient-reported outcome measure and is ready for the assessment of the health-related quality of life of patients with coronary artery disease in Korea. To establish the clinical validity of the K-MacNew, additional studies should be conducted to verify the validity and reliability of the K-MacNew in a number of participants, including those with various types of coronary artery disease.

## Background

Heart disease is the second leading cause of death among Koreans after cancer [1]. Coronary artery disease (CAD) is a typical chronic disease with an increased prevalence over the past decade worldwide, including in Korea [1, 2]. With the development of treatment regimens, such as coronary intervention or coronary artery bypass surgery, the survival rate and survival time of patients with CAD have improved [3, 4]. However, patients with CAD experience physical problems, such as chest pain, fatigue, and exhaustion, and emotional reactions, such as boredom, sadness, helplessness, and anger, even after diagnosis or treatment. Additionally, patients with CAD experience social problems, including adapting to their roles in the workplace and in their families. These negative experiences are associated with low health-related quality of life (HRQOL) in patients with CAD [5, 6].

Traditionally, prevalence and mortality rates have been used as outcome indicators for existing or new treatment regimens. However, the use of patient-reported outcomes, such as HRQOL, has been recently recommended [7]. HRQOL is an important concept for evaluating a patient’s overall perceived health status. It provides information about the burden of disease and its impact on health [5]. In the clinical field, HRQOL is more frequently measured than biochemical indicators when evaluating the effectiveness of rehabilitation for patients with CAD; additionally, HRQOL can be used when making decisions about treatment [6, 8, 9]. The advantages of disease-specific HRQOL measures are partly from achieving specificity by measuring the frequency and severity of specific symptoms, such as chest pain in patients with CAD. The MacNew Heart Disease HRQOL questionnaire (MacNew) has been used worldwide [6, 7, 9, 10].

The MacNew is a modified version of the Quality of Life after Myocardial Infarction [11] and Quality of Life after Myocardial Infarction-2 [12] questionnaires, developed for patients who have survived an acute myocardial infarction to assess their feelings about a range of issues and identified concerns. The MacNew is used to examine HRQOL in patients with CAD and to assess the effectiveness of various behavioral interventions to promote medication adherence, cardiac rehabilitation, and secondary prevention of CAD [6, 13]. The MacNew can be used to examine patients’ experiences with CAD, related treatment, and the impact of those experiences on their activities of daily living and physical, emotional, and social functioning [13].

The 36-item Short Form Health Survey Instrument (SF-36) is a generic tool commonly used to measure HRQOL in patients with CAD in Korea [14, 15]. Disease-specific QOL questionnaires, as the name suggests, tend to measure more specific elements of respective diseases; thus, they are theoretically more sensitive to subtle treatment-related changes than generic QOL measures [16]. The MacNew could provide a global HRQOL score by measuring the levels of physical, emotional, and social functioning in patients with CAD and offer a holistic understanding of the impact of the disease and treatment on an individual's life. Therefore, the MacNew is more appropriate than the SF-36 for measuring HRQOL in CAD patients.

The MacNew has been translated and validated in more than 20 languages, which are available on the MacNew website (macnew.org). The MacNew Questionnaire was linguistically adapted to the Korean population by Kang and colleagues [14]. However, Kang and colleagues [14] just tested its theoretical construct validity using partial confirmatory factor analysis for only 136 patients with myocardial infarction and identified five factors for the MacNew without a five-factor solution. Considering that the MacNew is often used worldwide as a patient-reported outcome for those with CAD, including those with myocardial infarction and unstable angina [6, 7, 9, 10, 17], it is necessary to evaluate the psychometric properties of the Korean version of the MacNew in Korean patients with CAD. Thus, this study was conducted to test the reliability and validity of the 27 items of the Korean version of the MacNew (K-MacNew).

## Methods

### Design

This study used a descriptive design to ensure that the translation of the MacNew into Korean was methodologically sound for use with Korean patients with CAD.

### Participants

A convenience sample was selected, comprising 205 participants who visited the cardiovascular clinic of a tertiary medical center in South Korea. The inclusion criteria were as follows: (a) diagnosed with unstable angina pectoris or myocardial infarction by a cardiologist, (b) aged over 19 years, (c) able to understand and speak Korean, (d) did not take drugs related to the central nervous system, (e) lived in Korea, and (f) able to understand the study and provide informed consent.

Of the 205 participants who answered the questionnaire, five were excluded owing to missing or incomplete informational responses. Thus, data from 200 participants were included in the final analysis.

### Instruments

#### The MacNew heart disease HRQL questionnaire

The MacNew was translated in accordance with the method suggested by the MacNew organization. Permission was obtained for psychometric testing of the MacNew from MacNew.org and the tool’s developer. In the first step, two independent translators (one health professional and one nursing professor), who were fluent in Korean and English and understood the cultural differences between Korea and the US, translated it into Korean. In the second step, two nursing professors analyzed the translated version to identify and revise items that needed correction due to issues related to translation accuracy and cultural differences, and then edited them to create one harmonized Korean version. In this step, for questions about the degree of experience with negative emotions in the past two weeks (Items 1 and 4), “how much” was revised to “how often,” to make it consistent with the response options for other questions. In the third step, the tool was independently back-translated into English by two bilingual translators (one nursing professor and one professional translator) unfamiliar with the original version. Finally, based on the English version reported by the original developer, the author reviewed and re-verified the Korean version to confirm the clarity and cultural relevance of the content. The final version of the K-MacNew was pilot tested using 10 patients with CAD who had never been exposed to the questionnaire. As all participants in this pilot test stated that the content was appropriate and easy to understand, the K-MacNew was finalized.

The MacNew includes 27 items in three dimensions: emotional, physical, and social. Each item is rated on a 7-point Likert scale ranging from “1 = poor HRQOL” to “7 = high HRQOL.” Some items are used more than once across the dimensions [12]. The global score is the mean score for all 27 items, wherein, each participant’s score is summed and divided by the total number of items [13]. The score for each dimension is the mean score for the items in that dimension, and omitted items are excluded from the calculation. Item 27, which is about the respondent’s sex life, can be excluded during scoring. The global and individual dimensional scores range from 1 to 7. The minimal important difference (MID) for the total MacNew and each dimension of the MacNew is 0.50 points [18]. Higher scores indicate higher HRQOL [13]. For the global MacNew, Cronbach’s α was 0.96 in Hofer and colleagues [13].

#### The 36-item short form health survey instrument (SF-36)

The SF-36 version 2 was used to assess concurrent validity. It is a self-administered questionnaire comprising 36 items with two components (physical and mental) [19]. The physical component summary includes four domains: physical role functioning, physical role, bodily pain, and general health. The mental component summary includes four domains: vitality, social functioning, emotional role, and mental health. This instrument uses a norm-based scoring method, in which the score for each domain is converted to a score out of 100, or the observed score is standardized with reference to the American population with a mean of 50 and standard deviation of 10 [20]. This method was used in the present study, and higher scores indicated higher HRQOL. The SF-36 version 2 has been extensively used internationally [19, 20], and the validity and reliability of the Korean version have been examined [21]. Cronbach’s α for the Korean SF-36 version 2 ranges from 0.82 to 0.94 for the physical health domain, and from 0.64 to 0.91 for the mental health domain [21]. Cronbach’s α in this study was 0.91 and 0.89 for the physical and mental health domains, respectively. For use in the present study, the questionnaire was licensed by Optuminsight Life Sciences, Inc. (Lincoln, RI, USA; License No: QM044578).

#### The hospital anxiety and depression scale (HADS)

The HADS was used to assess between-group discriminative validity [22]. It consists of 14 items, wherein the seven odd-numbered items are about anxiety, and the seven even-numbered items are about depression. Each item is rated on a 4-point scale ranging from 0 (no symptoms) to 3 (severe). Total scores range from 0 to 21, with higher scores indicating higher anxiety and depression. The validity and reliability of the Korean version of the HADS have been reported [23]. A cut-off score of 8 has been widely used internationally in clinical trials to classify patients with symptoms of anxiety and depression [22, 23]. Cronbach’s α was 0.86 for depression and 0.89 for anxiety in a previous study [23], and 0.77 for depression and 0.86 for anxiety in the present study.

#### The Korean activity scale index (KASI)

The KASI [24] is a self-report questionnaire measuring heart disease patients’ functional capacity. In the present study, the KASI was used to determine between-group discriminative validity. It includes 15 questions, and the score is multiplied by the weight of the response to each question to calculate the total score. Total possible scores range from 0 to 79, and higher scores indicate better functional capacity. Depending on KASI scores, classification of functional capacity ranges from Class 1 (more than 46 points) to Class 4 (less than 4 points). As a result of comparing the exercise load test to verify the validity of the KASI, the correlation between exercise time and the exercise load test was 0.62 (*P* < 0.001), and the correlation between the classification of functional capacity (calculated from the KASI) and the functional grade (calculated from the exercise load test) was 0.49 (*P* < 0.001) [24]. Cronbach’s α was 0.84 for the KASI in this study.

### Data collection

After obtaining ethical approval, data were collected in cooperation with the cardiology clinic from August 2018 to February 2019. To minimize participant dropout during the data collection process, research assistants distributed and collected the questionnaires by meeting in-person with those who voluntarily agreed to participate in this study in the cardiology outpatient clinic. Participants answered the survey in a quiet room, and it took approximately 15 min to complete.

### Statistical analysis

Analyses were performed using descriptive statistics and validity and reliability tests. Descriptive statistics were used to establish the analyzed general characteristics of the participants and the K-MacNew scores. For the validity assessment, construct and concurrent validity were evaluated. First, to assess construct validity, an exploratory factor analysis (EFA) was performed, and between-group discriminative validity was evaluated. Before performing the EFA, the Kaiser–Meyer–Olkin measure of sampling adequacy (KMO ≥ 0.5) [25] and Bartlett’s test of sphericity were used to determine whether the use of factor analysis was suitable. Principal component analysis (PCA) with varimax rotation, which has been frequently used in previous studies for factor analysis of the MacNew [6, 7, 26, 27], was performed to confirm the underlying factor structure of K-MacNew. The number of factors was determined based on the parallel test and eigenvalues > 1. Known-group methodology was used to examine discriminative validity [28] and to identify significant differences in the K-MacNew scores among functional capacity, anxiety and depression groups. An ANOVA with Bonferroni correction was performed to analyze the differences between the KASI classifications. We hypothesized that the K-MacNew scores would be lower in CAD patients with low functional capacity. Additionally, an independent t-test was performed to analyze differences in K-MacNew scores between groups according to anxiety and depression levels [6–8]. We hypothesized that the K-MacNew scores would be lower in CAD patients with anxiety and depression compared to those without.

Next, to assess concurrent validity, correlations were evaluated between the physical and emotional dimensions of the K-MacNew and the physical and mental health domains of the SF-36, based on previous studies [6, 29]. Pearson correlation coefficient analysis was used to test concurrent validity, and it was determined that coefficients from 0.1 to 0.3 would represent low correlations, 0.3 to 0.5 would be medium, and above 0.5 would be high. To assess reliability, internal consistency was tested using Cronbach’s α, with a coefficient of 0.70 interpreted as acceptable and 0.80 as high [30]. The collected data were analyzed using SPSS version 25 software (IBM, Armonk, NY, USA).

### Ethical statement

The present study was approved by the institutional review board of the authors’ affiliated university hospital (no. AJIRB-SBR-SUR-17–503). Prior to data collection, we informed the participants about the study purpose, the risks and benefits of participation, the principles of voluntary participation, the right to withdraw or refuse participation without consequences, and that collected data would be used for research purposes only. Only those who provided written consent were administered the questionnaire. Completed questionnaires were immediately placed in a sealed envelope and collected by a trained research assistant. The collected questionnaires were coded and entered into a computer upon completion of data collection and stored in a locked cabinet.

## Results

### Participants’ general characteristics

Mean participant age was 59.79 years. Most participants were men (85.0%), married (83.5%), employed (71.5%), and perceived economic level as “average.” Close to half of the sample (42.0%) had a university education or higher, and reported that they did not currently drink alcohol (45.5%); meanwhile most of the participants reported that they did not currently smoke (81.0%). The mean body mass index was 25.09 kg/m^2^, and approximately half of the sample (50.5%) were above 25 kg/m^2^. Of the 200 patients, 62.5% were diagnosed with unstable angina and 37.5% with myocardial infarction. According to the KASI classification, 71.5% of the participants were at Class 1, which indicates good functional capacity. Other relevant characteristics are shown in Table 1.

**Table 1:** Participants’ General Characteristics (N = 200)

Characteristics	Category	n (%)	Mean ± SD (range)
Age (years)	< 55	55 (27.5)	59.79 ± 9.46
	≥ 55–64	81 (40.5)	(21–78)
	≥ 65	64 (32.0)	
Sex	Men	170 (85.0)	
	Women	30 (15.0)	
Marital status	Single	33 (16.5)	
	Married	167 (83.5)	
Educational level	≤ Middle school	37 (18.5)	
	High school	79 (39.5)	
	≥ University	84 (42.0)	
Employment status	No	57 (28.5)	
	Yes	143 (71.5)	
Perceived economic level	Good	35 (17.5)	
	Average	137 (68.5)	
	Poor	28 (14.0)	
Current alcohol drinking	No	90 (45.0)	
	Yes	110 (55.0)	
Current smoking	No	162 (81.0)	
	Yes	38 (19.0)	
Body mass index	< 23	45 (22.7)	25.09 ± 2.69
	23–24.99	53 (26.8)	(17.65–36.33)
	≥ 25	100 (50.5)	
Diagnosis	Unstable angina	125 (62.5)	
	Myocardial infarction	75 (37.5)	
KASI class	Class I	143 (71.5)	55.87 ± 18.74
	Class II	42 (21.0)	(15.30–76.80)
	Class III	15 (7.5)	

KASI, Korean Activity Scale Index

### Korean version of MacNew scores

As a result of analyzing the K-MacNew scores, the mean of global, emotional, physical, and social scores were 5.98, 5.81, 5.98, and 6.24 points, respectively.

### Construct validity

The KMO value was 0.93 and Bartlett’s test of sphericity was χ^2^ (351) = 3419.434 (*P* < 0.001), indicating that the data were suitable for PCA. Three factors were identified in the parallel test, and four factors were identified with an eigenvalue > 1. As the original MacNew comprises three factors, the K-MacNew also included three factors. The communality of all 27 items was 0.3 or higher; therefore, no items were removed. The cutoff point used in the current study was a loading criterion of 0.40 [12, 31]. Twenty-seven items were cross-loaded on three factors: 15 items (1, 2, 3, 4, 5, 6, 7, 8, 10, 11, 12, 13, 15, 18, and 23) on Factor 1 (emotional dimension), 9 items (6, 9, 14, 15, 16, 17, 19, 20, 21) on Factor 2 (physical dimension), and 11 items (12, 13, 17, 20, 21, 22, 23, 24, 25, 26, and 27) on Factor 3 (social dimension). The explanatory power was 57.92% of the total variance (Table 2).

**Table 2:** Factor weights and variance explained from factor analysis (N = 200)

No	Item abbreviated descriptors	Emotional	Physical	Social	K-MacNew dimensions	MacNew dimensions
1	Frustrated	**0.712**	0.196	0.227	E	E
2	Worthless	**0.710**	0.192	0.211	E	ES
3	Confident	**0.502**	0.111	0.191	E	E
4	Down in the dumps	**0.793**	0.229	0.222	E	E
5	Relaxed	**0.640**	0.275	0.048	E	E
6	Worn out	**0.581**	**0.450**	0.268	EP	EP
7	Happy with personal life	**0.681**	0.249	-0.024	E	E
8	Restless	**0.654**	0.308	0.244	E	E
9	Shortness of breath	0.221	**0.725**	0.253	P	P
10	Tearful	**0.692**	0.100	0.267	E	E
11	More dependent	**0.624**	0.206	0.343	E	S
12	Social activities	**0.480**	0.230	**0.500**	ES	EPS
13	Others/less confidence in you	**0.512**	0.115	**0.420**	ES	ES
14	Chest pain	0.274	**0.709**	0.168	P	P
15	Lack self-confidence	**0.582**	**0.493**	0.255	EP	ES
16	Aching legs	0.202	**0.644**	0.190	P	P
17	Sports/exercise limited	0.182	**0.455**	**0.593**	PS	PS
18	Frightened	**0.612**	0.370	0.368	E	E
19	Dizzy or lightheaded	0.396	**0.530**	0.122	P	P
20	Restricted or limited	0.321	**0.451**	**0.586**	PS	PS
21	Unsure about exercise	0.213	**0.509**	**0.419**	PS	PS
22	Overprotective family	0.219	0.299	**0.609**	S	S
23	Burden on others	**0.488**	0.126	**0.525**	ES	ES
24	Excluded	0.267	0.170	**0.781**	S	PS
25	Unable to socialize	0.192	0.153	**0.747**	S	PS
26	Physical activity	0.210	0.315	**0.772**	S	PS
27	Sexual activity	0.081	0.045	**0.731**	S	P
	Variance explained	**24.26%**	**18.86%**	**14.80%**		

E, Emotional; P, Physical; S, Social; K-MacNew, The Korean version of the MacNew

Factor loading (loading ≥ 0.40), for each MacNew item in the Korean version (in bold); together with the factor loadings (loading ≥ 0.40) from the original factor analysis (in bold)

Between-group discriminative validity was determined by comparing the mean scores of the K-MacNew according to the KASI, anxiety and depression groups (Table 3). The results showed that the mean global score was significantly higher in KASI Class 1 than KASI Class 2 or Class 3 (*F* = 20.74, *P* < 0.001). Mean scores in all three dimensions were significantly higher in KASI Class 1 than KASI Class 2 or Class 3. The mean differences between KASI Class 1 and Class 2 and between KASI Class 1 and Class 3 were significantly higher than the established MID of 0.5. In addition, the global mean scores and those of the three dimensions were significantly lower in patients with anxiety and depression. Mean differences between CAD patients with anxiety and depression were higher than the established MID of 0.5.

**Table 3:** Differences in mean scores for the K-MacNew by KASI, Anxiety, and Depression Classification (N = 200)

Characteristic	Global Mean ± SD	Emotional Mean ± SD	Social Mean ± SD	Physical Mean ± SD
KASI class				
Class I (n = 143)^a^	6.19 ± 0.73	6.04 ± 0.84	6.46 ± 0.71	6.22 ± 0.78
Class II (n = 42)^b^	5.56 ± 0.88	5.33 ± 0.98	5.84 ± 1.02	5.54 ± 0.98
Class III (n = 15)^c^	5.16 ± 0.73	5.05 ± 0.93	5.25 ± 0.95	4.94 ± 0.88
F (*P*)	20.74 (< 0.001)	16.98 (< 0.001)	22.29 (< 0.001)	23.43 (< 0.001)
Post-hoc analysis	a > b,c	a > b,c	a > b > c	a > b,c
Anxiety				
No (n = 158)	6.21 ± 0.69	6.10 ± 0.74	6.41 ± 0.80	6.18 ± 0.79
Yes (n = 42)	5.12 ± 0.80	4.75 ± 0.87	5.58 ± 0.91	5.23 ± 0.99
t (*P*)	8.82 (< 0.001)	10.18 (< 0.001)	5.84 (< 0.001)	6.57 (< 0.001)
Depression				
No (n = 143)	6.28 ± 0.62	6.16 ± 0.70	6.48 ± 0.69	6.28 ± 0.67
Yes (n = 57)	5.25 ± 0.87	4.96 ± 0.92	5.63 ± 1.03	5.22 ± 1.02
t (*P*)	8.14 (< 0.001)	8.85 (< 0.001)	5.79 (< 0.001)	7.26 (< 0.001)

### Concurrent validity

Concurrent validity was tested by comparing correlations between the physical and emotional dimensions of the K-MacNew and the physical and mental components of the SF-36 (Table 4). According to the results of the analysis, the K-MacNew’s physical score showed a medium-sized correlation with the SF-36’s physical component (r = 0.517, *P* < 0.001), and the K-MacNew’s emotional dimension showed a strong correlation with the SF-36’s mental component (r = 0.745, *P* < 0.001). Thus, concurrent validity was established (Table 4).

**Table 4:** Correlations between the K-MacNew and SF-36 domains (N = 200)

Variable	Global r (*P*)	Emotional r (*P*)	Social r (*P*)	Physical r (*P*)
SF-36				
Physical component	0.493 (< 0.001)	0.434 (< 0.001)	0.490 (< 0.001)	0.517 (< 0.001)
Mental component	0.726 (< 0.001)	0.745 (< 0.001)	0.582 (< 0.001)	0.646 (< 0.001)

K-MacNew, The Korean version of the MacNew; SF-36, Short Form-36

### Internal consistency

Cronbach’s α for the K-MacNew were acceptable, with 0.947 for the global, 0.928 for emotional, 0.903 for social, and 0.928 for physical dimensions.

## Discussion

The MacNew is a questionnaire to assess HRQOL in patients with CAD and comprises three dimensions: physical, emotional, and social [7]. Since being validated at the time of development for use in patients with heart disease, it has been translated from English into several other languages (macnew.org). In this study, we tested the construct validity, concurrent validity, and internal consistency reliability of the K-MacNew to determine whether the instrument can be used among patients with CAD in Korea.

As the MacNew comprises three dimensions, we also performed an EFA to verify the construct validity of the K-MacNew. Generally, an eigenvalue greater than 1 has been used as the criterion to determine the number of factors in factor analysis in studies testing the construct validity of multidimensional instruments. However, owing to concerns that this may overestimate the number of factors, parallel tests have been performed in recent studies to determine the appropriate number of factors [32]. Hence, we determined that the K-MacNew has three factors based on theoretical evidence, PCA with varimax rotation, and a parallel test. The EFA yielded an explanatory power of 57.92% of the total variance.

The MacNew was used with a factor loading threshold of > 0.40 in the factor structure analysis [12, 31]. Therefore, as the MacNew allows cross-loading of items, one item is often cross-loaded onto different factors during factor analysis to test construct validity. The original tool has 12 items that are cross-loaded onto two or more factors [31]. Furthermore, although it varies across studies, previous studies reported that at least three items are cross-loaded onto two or more factors [6, 7, 10, 26]. The K-MacNew also features seven items that are cross-loaded onto two factors, and one item is cross-loaded onto three factors. However, some researchers have questioned the validity of including all items with a factor weight > 0.40 [10, 33]. This is because the results vary across studies, in which 27 items are loaded onto different factors depending on the language, and some items are loaded onto dimensions not explained in the original instrument.

Of the 27 items included in this study, 15 were loaded onto the emotional dimension. All 14 items from the emotional dimension of the original MacNew were included in the emotional dimension of the Korean version, along with one additional item (Item 11, more dependent). Item 11 was loaded onto the social dimension in the original version and the physical dimension in the Sri Lankan version [10], and was not loaded onto any of the three dimensions in the Chinese [6] and Turkish versions [27]. The factor loadings of Item 11 across studies may be attributable to the cultural differences that occur when interpreting the statements of the instrument. In other words, in Korean patients, becoming more physically dependent owing to a heart condition may have been perceived as becoming an emotional burden for significant others, such as one’s family. Of the 15 items, the loading value was the highest for Item 4 (down in the dumps), followed by Items 1 (frustrated), 2 (worthless), and 10 (tearful). This is similar to the results of a previous study in which the emotional dimension addressed emotional responses generally experienced by patients with CAD, such as depression [26].

Nine out of the 27 items were loaded on the physical dimension. Of these items, eight were consistent with the original MacNew, and Item 15 (lack self-confidence) was added. Item 15 was cross-loaded onto the emotional and social dimensions in the original version, but cross-loaded onto the emotional and physical dimensions in the Korean version. This may be attributable to the ambiguity of the original tool. For instance, Item 3—loaded onto the emotional dimension—specifically asks about one’s confidence and decision-making regarding the management of heart problems. However, Item 15 asks about overall lack of confidence, without specifying to what that confidence is related. Therefore, it is possible that participants understood Item 15 to be asking about self-confidence in symptom management; however, considering that Item 15 was cross-loaded onto the emotional and social dimensions in previous studies [6, 7, 10], it is necessary to conduct replication studies with a larger sample to further confirm the construct validity of the K-MacNew.

The physical dimension assesses the level of physical symptoms of heart disease and resulting physical limitations. The items with the highest loading values for the physical dimension in our study were items pertinent to physical symptoms (Item 9: shortness of breath, Item 14: chest pain, Item 16: aching legs, and Item 19, dizzy or lightheaded). However, five items that had been loaded onto the physical dimension in the original instrument (Items 12, 24, 25, 26, and 27) were instead included in the social dimension in our study. This was similar to the versions from other Asian countries, including the Sri Lankan [10] and Chinese versions [6]. This suggests that patients perceive items related to physical limitations to be pertinent to engaging in less social activity as a result of such limitations. Thus, subsequent studies should make multinational comparisons of how items related to physical symptoms are perceived among patients with CAD in various Asian countries.

Finally, 11 of the 27 items of the K-MacNew were loaded onto the social dimension. Ten of these were consistent with the original, and Item 27 (sexual activity) was included. A number of previous studies excluded Item 27 when testing construct validity owing to cultural reasons [13]. However, sexual activity is important to consider when examining the HRQOL of patients with heart disease, as it can affect one’s physiological needs and intimate relationship with the spouse. Item 27, which was classified into the physical dimension originally, was loaded into the social dimension in this study. This is similar to the results of a previous study that tested the construct validity of the English version of the MacNew in patients with angina and ischemic heart failure [13]. The loading of Item 27 onto the social dimension is related to the fact that this dimension of the MacNew assesses social situations experienced in a physical or emotional context [13]. However, it is necessary to confirm the appropriateness of the K-MacNew factor loadings via a confirmatory factor analysis. In the present study, Items 24 (excluded), 26 (physical activity), and 25 (unable to socialize) had the highest factor loadings in the social dimension. These items also had high factor loadings and were cross-loaded onto the physical and social dimensions in the original version. However, these items were only loaded onto the social dimension in this study, presumably owing to cultural differences in how disease impact was perceived. Therefore, participants seemed to perceive their physical symptoms as physical health problems and disease-related limitations to social and other activities as social health problems.

Between-group discriminative validity was tested with the KASI, anxiety and depression levels of CAD patients. The KASI Class 1 group, which indicates good functional capacity, scored significantly higher on the K-MacNew compared to the KASI Classes 2 and 3. Moreover, the MID of the K-MacNew score between the groups, especially between KASI Class 1 and Classes 2 and 3, exceeded 0.5 [18]. This is similar to the findings of a previous study on patients with ischemic heart disease, which reported that the good functional status group obtained significantly higher MacNew scores compared to the poor functional status group, and the difference was higher than the MID [8]. Furthermore, the K-MacNew scores were lower in CAD patients with anxiety and depression than those without. This is consistent with previous reports that HRQOL decreases as depression and anxiety increase [6–8]. Consequently, the K-MacNew would be a useful instrument in clinical settings to examine HRQOL according to the level of physical activity and anxiety and depression in patients with CAD, as well as to assess the effects of cardiac rehabilitation therapies.

To test the concurrent validity of the K-MacNew, we analyzed the physical and emotional dimensions of the K-MacNew and the physical health and mental health domains of the SF-36, respectively. The correlation coefficients ranged from 0.53 to 0.75, showing a moderate correlation. This finding is in line with prior studies [7, 13].

Regarding internal consistency reliability, Cronbach’s α ranged from 0.88 to 0.93, and Cronbach’s α for the global and dimensional scores were similarly high. This is similar to the level of internal consistency reported by previous studies that applied the MacNew in Asia [6, 10]. The K-MacNew is a homogeneous tool for measuring HRQOL in patients with CAD in Korea. However, we could not verify its test–retest reliability; therefore, subsequent studies to ensure scoring stability in addition to item homogeneity are needed.

This study had a few limitations. As participants were selected using convenience sampling, the sample is not representative of all patients with CAD. Since this study did not include elderly participants over 80 years of age, it is necessary to conduct follow-up studies in the future by expanding the age range for participants to ensure the validity and reliability of K-MacNew. Second, most participants were men and diagnosed with unstable angina; thus, caution should be taken when interpreting the validity of the K-MacNew with women and those with myocardial infarction. Although unstable angina and myocardial infarction are both classified as acute coronary syndromes, the pathophysiological mechanisms and prognosis of the two diseases differ. Therefore, a follow-up study is needed to investigate the feasibility of the K-MacNew by recruiting sufficient participants for each disease group. Despite these limitations, this study is significant in that it helps to cross-culturally validate the MacNew. Therefore, accumulating further evidence on the validity and reliability of the K-MacNew through replication studies would enhance the sensitivity of the K-MacNew to assess HRQOL in patients with CAD and promote its wide use in clinical settings.

## Conclusions

We systematically translated the MacNew—an HRQOL tool widely used in international studies—into Korean and validated it in patients with CAD in Korea. The K-MacNew had established construct validity and criterion validity. It was also highly reliable; therefore, the K-MacNew is a validated instrument for measuring HRQOL in patients with CAD. Based on our findings, the K-MacNew would be useful in developing and assessing intervention programs to enhance HRQOL in patients with CAD in Korea.

## Data Availability

All data generated or analyzed during this study are included in this published article.

## Abbreviations

CAD: Coronary artery disease
EFA: Exploratory factor analysis
HADS: The Hospital Anxiety and Depression Scale
HRQOL: Health-related quality of life
K-MacNew: Korean version of the MacNew Heart Disease HRQOL questionnaire
KASI: The Korean Activity Scale Index
KMO: Kaiser–Meyer–Olkin
MacNew: The MacNew Heart Disease HRQOL questionnaire
PCA: Principal component analysis
SF-36: The 36-Item Short Form Health Survey Instrument
